# Quantitative evaluation of scar, area at risk, and wall thickening in a porcine model of sub-acute myocardial infarction (MI)

**DOI:** 10.1186/1532-429X-15-S1-P213

**Published:** 2013-01-30

**Authors:** Ramkumar Krishnamurthy, Ke Li, Amol Pednekar, Benjamin Cheong, Raja Muthupillai

**Affiliations:** 1 Radiology, St. Luke's Episcopal Hospital, Houston, TX, USA; 2 Philips Health Care, Houston, TX, USA

## Background

Elevated signal intensity (SI) in T2w images is often designated as area-at-risk (AAR) following acute/sub-acute MI. While AAR has been qualitatively associated with reductions in wall thickening (WT), quantitative information between the two is not available [1-3]. Quantitative SI threshold for classifying regions as AAR, and its effect on regional WT have also been not reported.

## Purpose

In an animal model, quantify the:

1) Effect of SI threshold on estimating AAR,

2) Reduction in WT with AAR at increasing SI thresholds, and

3) Correlation between AAR and percent scar.

## Methods

Acquisition Protocol: Basal, mid, and apical slices in short axis orientation were obtained in a pig (n=14) AMI model (LAD occlusion) at 3.0T (Ingenia, Philips Healthcare):

a) Cine SSFP: TR/TE/α = 3/1.5 ms/45°; acquired temporal resolution: 12 ms.

b) Delayed enhancement MRI (DE-MRI): 10 min after 0.2 mmol/kg of contrast, scar was visualized using a gated IR-TFE sequence with inversion delay (TI) adjusted to null normal myocardium.

c) Dual-IR T2W imaging (BB): Effective TE/TR: 80 ms/2*RR interval; TSE readout.

**Figure 1:**
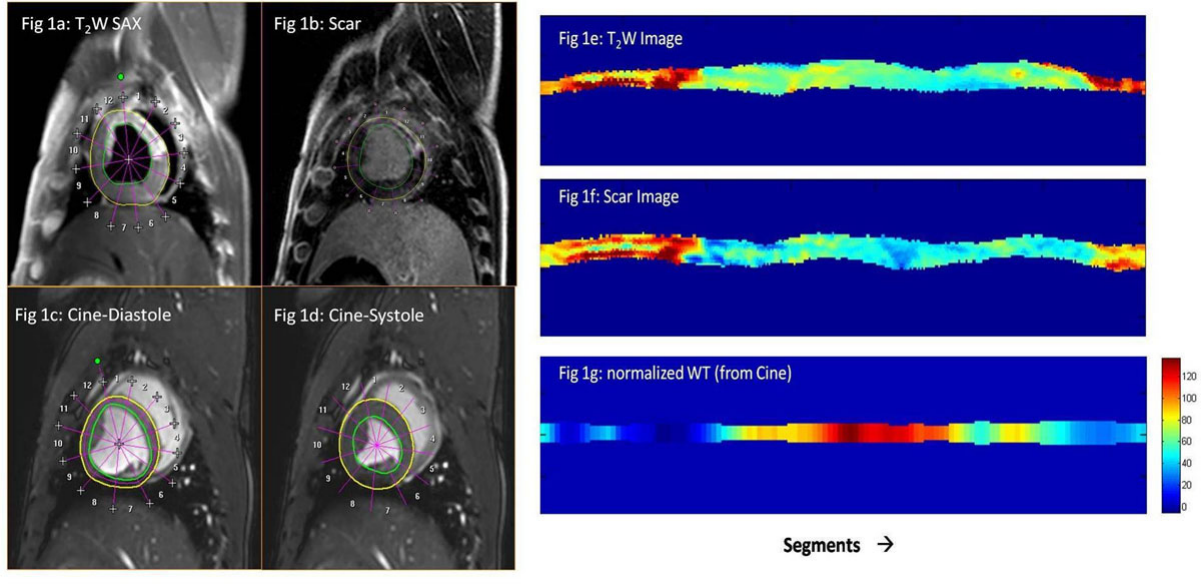
The short axis images of the LV is divided into multiple segments for the T_2_Weighted(a), scar(b) and Cine (c and d) images. From the cine image, normalized wall thickness is also obtained (nWT). Polar plot of the same are obtained (e, f and g) after post proecssing.

Data Analysis: Myocardial region was manually segmented from cine, DE-MRI, and BB images (Figure 1) using MATLAB™. Myocardial region was sub-divided into co-registered segments of equal mass for each animal to calculate the following quantitative parameters:

1) Normalized wall thickness (nWT) per segment = (WTES - WTED)/( WTED), where ED = End-Diastole, ES-End-Systole.

2) Segmental scar burden, defined as ratio of pixels designated as scar to total number of pixels in each segment of DE-MRI. Scar pixel is one with SI > [mean + 5*Standard Deviation (SD) of normal remote myocardium].

3) Segmental AAR, defined as regions in BB images with SI > mean + n*SD of normal remote myocardium (AAR with n = 2, 3, 4 are AAR_2SD, AAR_3SD, and AAR_4SD, respectively).

## Results

1) As a share of total myocardium, AAR burden was significantly higher (43 to 57 %) than scar burden (30 %) (Table 1). Although total AAR burden progressively declined with increasing T2w SI cut-offs, it was not lower than scar burden at any threshold evaluated (AAR_4SD > Scar burden).

**Table 1:** AAR and scar burden calculated for LV, expressed as a percentage of total myocardial mass. AAR Burden at different quantifying metric is consistently greater than scar burden (p<0.01).

AAR_2SD	AAR_3SD	AAR_4SD	Scar
57 ± 6.4%	49.8 ± 6.8%	43.6 ± 8%	30.2 ± 14.7%

**Figure 2:**
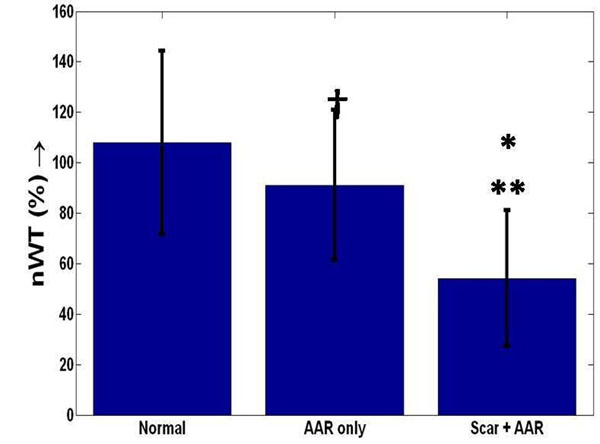
Variation of normalized wall thickening (nWT) for three difference regions of the myocardium. 1) Normal region corresponds to no scar or AAR, 2) AAR only are regions that have no scar but only AAR, 3) Scar + AAR has both scar and AAR present. AAR_SD2 is shown. †→p = NS b/w Normal, AAR *, ** → p<0.002 b/w Scar and AAR and a) Normal and b) AAR only

2) Spatially, AAR overlapped, and extended beyond scar regions.

3) Reduction in segmental nWTwas lower in AAR regions that did not overlap scar region compared to those segments that did(108 ± 36 % vs 91 ± 29 %, Figure 2).

## Conclusions

In sub-acute AMI, AAR is significantly larger than scar. In non-overlapping regions of AAR and scar, nWT, while diminished compared to normal remote myocardium, is significantly better than in regions of scar.

## Funding

NA
